# Quality of CAD-CAM inlays placed on aged resin-based composite restorations used as deep margin elevation: a laboratory study

**DOI:** 10.1007/s00784-022-04841-y

**Published:** 2023-01-09

**Authors:** Catherine E. R. Theisen, Julia Amato, Gabriel Krastl, Thomas Attin, Markus B. Blatz, Roland Weiger, Florin Eggmann

**Affiliations:** 1 Department of Periodontology, Endodontology, and Cariology, University Center for Dental Medicine Basel UZB, University of Basel, Mattenstrasse 40, CH-4058 Basel, Switzerland; 2 Department of Conservative Dentistry and Periodontology, Center of Dental Traumatology, University Hospital of Würzburg, Würzburg, Germany; 3 Department of Conservative and Preventive Dentistry, Center of Dental Medicine, University of Zurich, Zurich, Switzerland; 4 Department of Preventive and Restorative Sciences, Penn Dental Medicine, University of Pennsylvania, Philadelphia, PA USA

**Keywords:** Cervical margin relocation, Composite resins, Dental caries, Dental restoration, Proximal box elevation, Subgingival margin

## Abstract

**Objectives:**

To assess the impact of the age of resin-based composite (RBC) restorations used for deep margin elevation (DME) on the marginal quality and fracture resistance of inlays.

**Materials and methods:**

Permanent human molars with direct RBC restorations, involving the mesial, occlusal, and distal surface (MOD), were allocated to four groups (each *n* = 12). Half of the teeth underwent thermomechanical loading including 240,000 occlusal load cycles and 534 thermal cycles (TML, 5 °C/55 °C; 49 N, 1.7 Hz). With RBC left in one proximal box as DME, all teeth received MOD inlays, made from lithium disilicate (LDS) or a polymer-infiltrated ceramic network material (PICN). The restored teeth underwent TML including 1.2 million occlusal cyclic loadings and 2673 thermal cycles. The marginal quality was assessed at baseline and after both runs of TML. Load-to-fracture tests were performed. The statistical analysis comprised multiple linear regressions (*α* = 0.05).

**Results:**

Simulated aging of RBC restorations had no significant effect on the marginal quality at the interface between the RBC and the tooth and the RBC and the inlay (*p* ≥ 0.247). Across time points, higher percentages of non-continuous margin were observed between the inlay and the tooth than between the tooth and the RBC (*p* ≤ 0.039). The age of the DME did not significantly affect the fracture resistance (*p* ≥ 0.052).

**Conclusions:**

Artificial aging of RBC restorations used for DME had no detrimental effect on the marginal quality and fracture resistance of LDS and PICN inlays.

**Clinical relevance:**

This laboratory study suggests that—in select cases—intact, direct RBC restorations not placed immediately before the delivery of an indirect restoration may be used for DME.

## Introduction

Restoration of teeth with subgingival defects remains a challenge. To restore such teeth with an indirect restoration, a direct restoration that relocates the cervical margin to a supragingival position is frequently used as a more straightforward alternative to surgical crown lengthening or orthodontic or surgical extrusion [1–3]. This restorative approach, termed deep margin elevation (DME), facilitates the subsequent preparation, impression taking, isolation with rubber dam, and adhesive luting of an indirect restoration [1]. According to the most recent review article, well-polished DME restorations made with proper isolation of the working field and not infringing on the supracrestal connective tissue attachment are compatible with periodontal health [1].

However, the number of clinical studies investigating DME is limited. Consequently, the available evidence on DME is largely based on laboratory studies [1]. The marginal quality of dental restorations is of paramount importance, as poor adaptation and defects promote the accumulation of biofilm, which may in turn cause periodontal disease, secondary caries, or both. Employing methods such as microscopic marginal quality assessments, microleakage measurements, and microcomputed tomography imaging, some of the laboratory studies evaluating the marginal quality of indirect restorations on DME made from resin-based composite (RBC) found the marginal adaptation to be inferior to that of indirect restorations directly bonded on dentin [4–6]. Yet, among in vitro studies, investigations reporting no such negative effect of DME currently prevail in number [7–14].

From a clinical perspective, it would sometimes be advantageous if parts of an existing RBC restoration could be kept for DME because this would eliminate the need to replace RBC that extends below the cementoenamel junction (CEJ) before an indirect restoration is delivered [15, 16]. Performing the procedure in this manner would, for example, simplify the provision of indirect restorations for posterior teeth with an intact RBC buildup restoration after endodontic treatment.

A recentin vitro study analyzed the performance of different RBC materials in deep proximal boxes, showing more favorable results for nanohybrid and bulk-fill RBC compared with flowable RBC [17]. But the body of laboratory evidence on DME consists mostly of studies that did not expose RBC restorations used for DME to any form of aging before bonding an indirect restoration on top. A review published in 2022 included 16 in vitro studies assessing the marginal quality of indirect restorations in teeth with DME [1]. In 11 out of these 16 studies, indirect restorations were made immediately after DME, while two and three studies reported water storage periods of 1 week at 37 °C and 2 weeks at room temperature, respectively [1]. None of these 16 studies subjected DME restorations to mechanical loading prior to the fabrication of indirect restorations [1]. It is therefore unclear whether using an existing direct RBC restoration for DME is a viable therapeutic approach.

Given this paucity of evidence, the aim of this laboratory study was to compare the marginal quality and fracture behavior of inlays, made from lithium disilicate (LDS) and a polymer-infiltrated ceramic network material (PICN), placed on artificially aged RBC restorations or RBC restorations without any aging.

## Materials and methods

### Ethical approval

The study complied with the regulatory requirements of the Swiss Human Research Act and Human Research Ordinance. The local ethics committee approved the use of irreversibly anonymized teeth from donors who provided informed consent to the use of their extracted tooth/teeth for research purposes (EKNZ UBE-15/111).

### Sample size calculation

An a priori sample size calculation was made using data on marginal quality reported in a previous in vitro study assessing DME restorations [8]. The type I and type II error rates were set at 0.05 (two-tailed) and 0.2, respectively. The effect size was set at 12.5%, the average of statistically significant differences in marginal quality across the four groups, and the standard deviation was set at 10.3%, the average of standard deviations calculated based on the 95% confidence intervals reported for said groups. With these parameters, the *t*-statistic resulted in a recommended sample size of 12 specimens per group.

### Specimen preparation

**Fig. 1:**
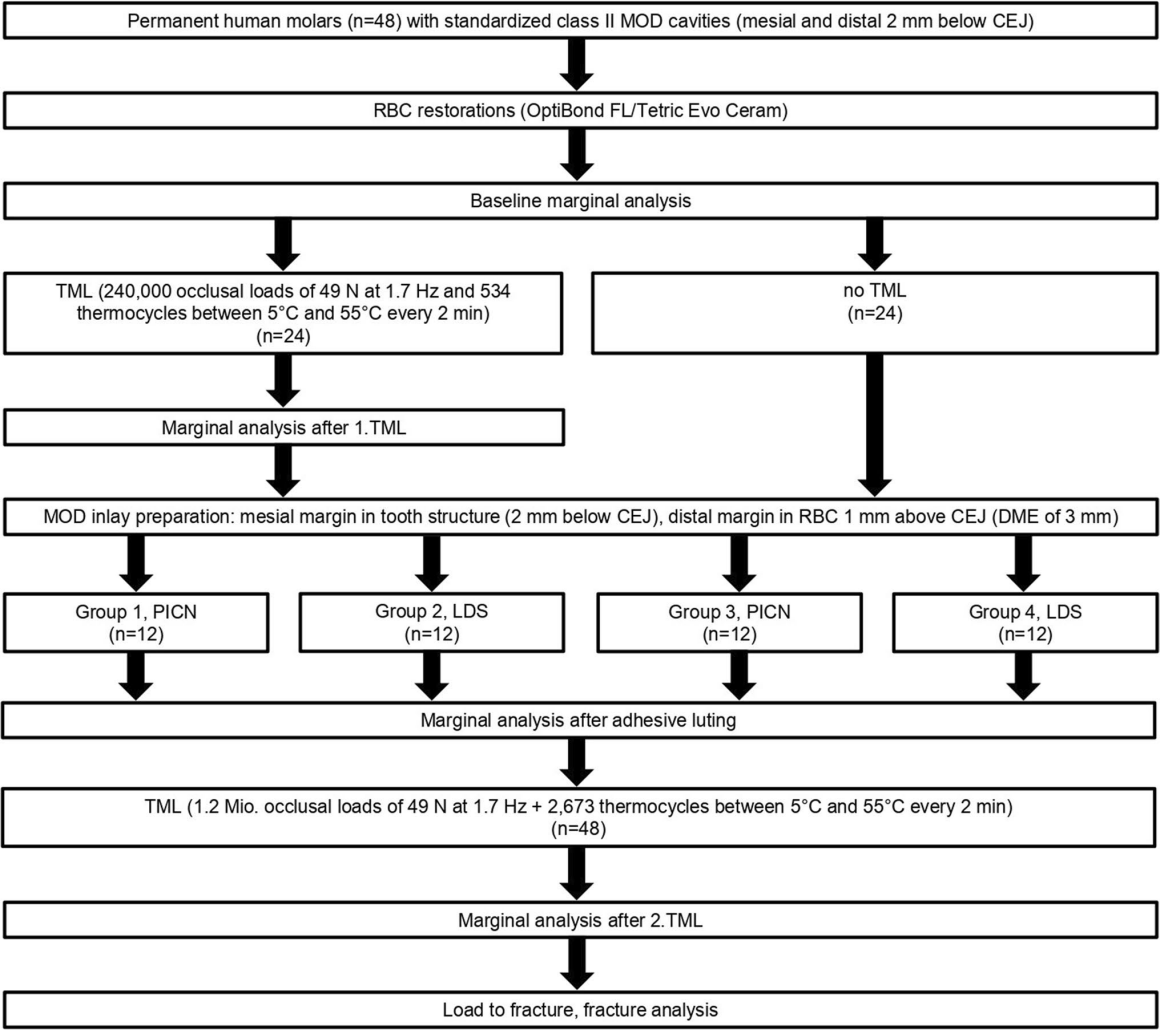
Flow chart with an overview of the study design

An overview of the study flow is shown in Fig. 1. Forty-eight permanent human molars of nearly equal size were cleaned after extraction with a universal curette and low-abrasive air polishing (Airflow Plus, E.M.S. Electro Medical Systems, Nyon, Switzerland). No effort was made to completely remove root cementum. The teeth, free of caries, restorations, craze lines, cracks, and significant signs of tooth wear, were stored in a 0.1% thymol solution until further processing.

One operator prepared standardized, box-shaped mesio-occluso-distal (MOD) cavities with water-cooled diamond burs (Intensiv Profi Prep Set Ref. 122, Intensiv, Montagnola, Switzerland), regularly checking the dimensions of the cavities with 3.5 × magnification loupes and a periodontal probe. The occlusal box was 2 mm deep and 3 mm wide. The proximal boxes reached 2 mm below the CEJ. They were 3.5 mm wide and 2 mm wide in the buccolingual and mesiodistal dimensions, respectively. Loose enamel rods were removed. The cavities featured no undermined enamel, and the margins were not beveled [18]. The operator secured a circumferential, metal matrix (Matrices Anatomiques [0.03 mm, Ref. 5718], Polydentia, Mezzovico-Vira, Switzerland) around each tooth with a Tofflemire retainer (Omni Matrizenspanner, Omnident, Rodgau Nieder-Roden, Germany), adapting the matrix with a Heidemann spatula to ensure a tight seal at the proximal cavity margins. The cavities were conditioned with a phosphoric acid etchant (Ultra-Etch, Ultradent Products, South Jordan, UT, USA) for 15 s, and an etch-and-rinse adhesive was applied according to the manufacturer’s instructions (OptiBond FL [Primer, Lot No. 7220095; Adhesive, Lot No. 7498497], Kerr Italia, Scafati, Italy). Light curing was performed for 20 s at an irradiance of 1200 mW/cm^2^ (Bluephase Style 20i [high power mode], Ivoclar, Schaan, Liechtenstein). Using the centripetal buildup technique, the pretreated class II cavities were restored with RBC (Tetric EvoCeram [A4, Lot No. Y37695], Ivoclar) [4, 8, 18]. Each increment, 2 mm thick or less, was light cured for 10 s at an irradiance of 1200 mW/cm^2^ (Bluephase Style 20i [high power mode], Ivoclar). The restoration was post-cured for 20 s from the lingual side and 20 s from the buccal side with the same curing light after the matrix had been removed. During light curing of the adhesive and the RBC, the distance between tip of the curing light and adhesive/RBC was kept as short as possible without contact to uncured material. After removal of excess material with surgical scalpels (No. 12D, Gebrüder Martin, Tuttlingen, Germany), the restoration margins were finished and polished with contouring and polishing discs (Sof-Lex, 3M, Saint Paul, MN, USA) and rotary polishing instruments (Kenda, Coltène/Whaledent, Altstätten, Switzerland) under an operating microscope (OPMI Pico, Carl Zeiss Meditec, Jena, Germany) at a magnification of 10 × .

To simulate the periodontal ligament, the roots up to 5 mm below the CEJ were covered with a 0.1–0.2-mm-thick layer of silicone (Affinis light body, Coltène/Whaledent) [19]. They were then embedded in epoxy casting resin (RenCast CW 20, OBO-Werke, Stadthagen, Germany) in a manner that the cervical restoration margins were 3 mm above the simulated alveolar bone level [8].

### Baseline marginal analysis

**Fig. 2:**
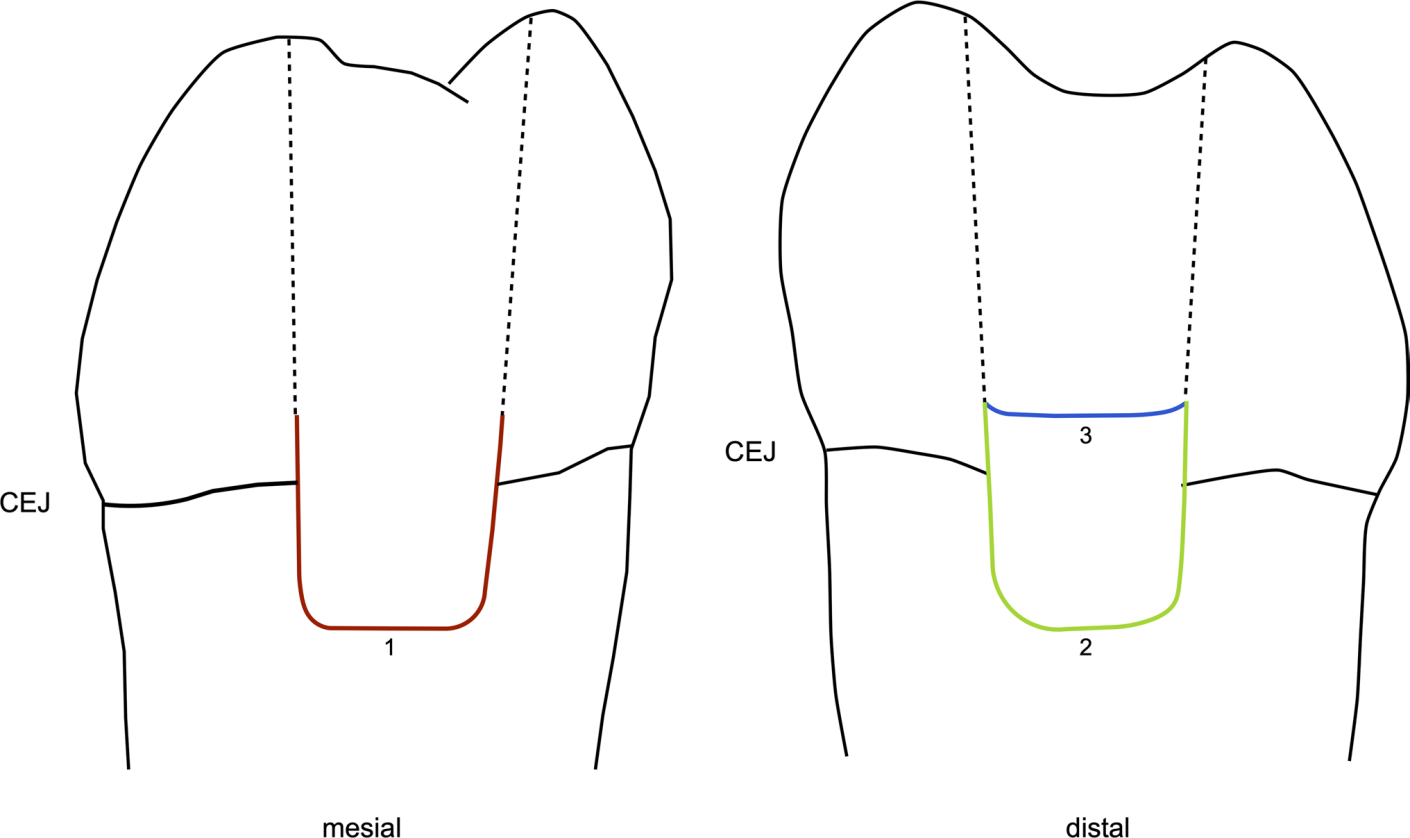
Schematic drawing of the proximal interfaces where the marginal integrity was assessed. The interface 1 (“tooth-inlay,” red line) was located on the mesial surface between the inlay and the tooth substance up to 1 mm above the CEJ. The interface 2 (“tooth-RBC,” green line) was located on the distal surface between the RBC left as DME and the tooth substance. The interface 3 (“RBC-inlay,” blue line) was located on the distal surface between the RBC left as DME and the inlay

Images of the proximal surfaces of the specimens were captured at a magnification of 20 × with a laser microscope (WD 11 combined with VKX-1000 3D Laser Scanning Microscope, Keyence, Mechelen, Belgium), which was used for image acquisition throughout the study. To ensure the images were acquired in a standardized manner, custom holders made from silicone-based putty (Coltoflax, Coltène/Whaledent) fixed the specimens on the stage of the microscope. Image size calibrations and quantitative marginal integrity evaluations were performed with image analysis software (Fiji) [20]. The integrity of the proximal margins of the RBC restorations was assessed up to 1 mm above the CEJ (Fig. 2). The marginal quality was classified as “continuous” (no gap), “non-continuous” (gap, interruption of continuity, or fractures related to restoration margins), or “not assessable/artifact” [8, 21].

### First thermomechanical loading (1.TML)

The specimens were randomly allocated to four groups (G1 to G4), each comprising 12 specimens (Fig. 1). Specimens in groups G1 and G2 underwent artificial aging. In a custom, computer-controlled masticator, the specimens were exposed to mechanical and thermal stress for 240,000 occlusal load cycles with 49 N at 1.7 Hz. Zirconia spheres (Mühlmeier, Bärnau, Germany) with a diameter of 5 mm were used as antagonists. The specimens simultaneously underwent 534 thermocycles between of 5 °C and 55 °C with a 2-min dwell time and a 15-s transition time [22, 23]. Meanwhile, specimens in groups G3 and G4 were stored in tap water at room temperature [22, 23].

### Marginal analysis after 1.TML

After the first TML, the quantitative marginal assessment of the specimens in groups G1 and G2 was performed following the same protocol as for the baseline measurement.

### Inlay preparation

All 48 specimens received a standardized MOD ceramic inlay preparation, made with water-cooled diamond burs (Intensiv Profi Prep Set Ref. 122, Intensiv). The external form of the preparation featured an isthmus width of half of the intercuspal dimension (≥ 3 mm), a flowing margin, and unbeveled cavosurface margins of approximately 90°. The internal form featured smooth and rounded internal line angles, ≥ 2.5 mm of occlusal depth measured from the deepest point of the central groove and approximately 10° to 12° of axial wall divergence. RBC was removed completely in the mesial proximal box and occlusally. The proximal boxes were 3.5 mm wide and 2 mm wide in buccolingual and mesiodistal dimensions, respectively. Their gingival floor was perpendicular to the cavosurface. The cervical margin was located 2 mm below the CEJ in the mesial proximal box and 1 mm above the CEJ in the distal box, where RBC was left as deep margin elevation [8]. A fluorescence-aided identification technique, described in detail elsewhere, ensured complete removal of RBC in the occlusal and mesial box [15]. To facilitate detection of RBC to be removed, the operator used a fluorescence-inducing device that emits light with wavelengths around 405 nm (SiroInspect, Dentsply Sirona, York, PA, USA). The operator, wearing magnifying loupes (3.5 ×), used the fluorescence-inducing device without a filter. During preparation, the operator monitored the dimensions of the cavities as described above. Using an intraoral scanner software tool (Prepcheck, Dentsply Sirona, Bensheim, Germany), all preparations were evaluated and adjusted if improvements were necessary before inlay fabrication. The floor of the distal box, where RBC had been left as deep margin elevation, was grit blasted with aluminum oxide particles (50 µm) perpendicular to the surface from 10 mm for 5 s at a pressure of 0.15 MPa. The cavity was thoroughly rinsed with water spray for 15 s and gently air dried.

### Fabrication of inlays

Digital impressions of the inlay preparations were obtained with an intraoral scanner (Cerec Omnicam, Dentsply Sirona). For all specimens, MOD inlays were fabricated with computer-aided design and computer-aided manufacturing (CAD-CAM) (Cerec Omnicam, software 5.1.3; inLab MC XL, Dentsply Sirona). To design the restorations, the so-called biogeneric individual design mode was employed. The default settings were used for the parameters “radial spacer” and “marginal adhesive gap,” which were set to 120 µm and 60 µm, respectively. The mode of the grinding and milling unit was set to “high” for the level of detail. The inlays in groups G1-PICN-2TML and G3-PICN-1TML were made from PICN (Vita Enamic [4M2-HT, Lot No. 90210], VITA, Bad Säckingen, Germany), whereas inlays in groups G2-LDS-2TML and G4-LDS-1TML were made from LDS (IPS e.max CAD CEREC/inLab [HT A4, Lot No. Z01FDS], Ivoclar). The inlays were tried in after sprue removal, and minor adjustments were made if necessary. LDS inlays underwent crystallization firing (Programat CS, Ivoclar), while PICN inlays were polished (Vita Enamic Polishing Set, VITA).

### Luting procedure

Selective enamel etching was performed with 35% phosphoric acid gel (Ultra-etch, Ultradent) for 20 s. The etchant was rinsed off with water spray for 20 s before the cavities were gently dried with compressed air. Starting with the enamel, a universal adhesive (Adhese Universal, Ivoclar) was applied with a microtip applicator so that the whole inlay cavity was coated. The adhesive was lightly scrubbed into the tooth surface for 20 s. To evaporate the solvents, the adhesive was gently air-dried for approximately 5 s until an immobile, glossy film layer resulted. The adhesive was light cured for 10 s at an irradiance of 1200 mW/cm^2^ (Bluephase Style 20i [high power mode], Ivoclar).

The intaglio surfaces of the LDS and PICN inlays were etched for 20 s and 60 s, respectively, with 5% hydrofluoric acid (HF) (Vita Ceramics Etch [Lot No. 89140], VITA). After HF etching, the inlays were thoroughly rinsed with water spray and ultrasonically cleaned in ethanol (96%) for 180 s. Using a microtip applicator, a thin layer of a single-phase silane-coupling agent (Vita Adiva C-Prime [Lot No. 94700], VITA) was applied on the dry intaglio surface and let set for 10 s. A gentle stream of compressed air was blown over the surface to evaporate the solvent until the surface was no longer moist and appeared glossy.

A thin, even layer of a dual curing, resin-based luting material (Variolink Esthetic DC neutral, Ivoclar), mixed according to the manufacturer’s instruction in a mixing syringe dispenser, was applied on the intaglio surface of the inlay. The inlay was seated with even, firm finger pressure and a ball-shaped plugger was used to hold it in place throughout the luting procedure. The proper seat of the inlay was checked with a probe. Excessive luting material was carefully removed with foam pellets (Pele Tim, Voco, Cuxhaven, Germany) before the restoration margins were covered with glycerin gel (Liquid Strip [Lot No. Z01T0G], Ivoclar). Light curing was performed from the mesial, distal, buccal, oral, and occlusal sides for 20 s each at an irradiance of 1200 mW/cm^2^ (Bluephase Style 20i [high power mode], Ivoclar). Under an operating microscope (OPMI Pico, Carl Zeiss Meditec) at a magnification of 10 × , the restoration margins were finished with contouring and polishing discs (Sof-Lex, 3M) before final polishing (for LDS, OptraFine, Ivoclar; for PICN, Vita Enamic Polishing Set, VITA). One operator performed all restorative procedures throughout the study.

### Marginal analysis after adhesive luting

After insertion of the inlays, the marginal integrity was examined at a magnification of 20 × at three different interfaces (Fig. 2) and assessed according to the protocol used at baseline.

### Second thermomechanical loading (2.TML)

Using the same setup as in the 1. TML, all 48 specimens underwent 1.2 million occlusal cyclic loadings with 49 N at 1.7 Hz and 2673 thermal cycles [22].

### Marginal analysis after 2.TML

**Fig. 3:**
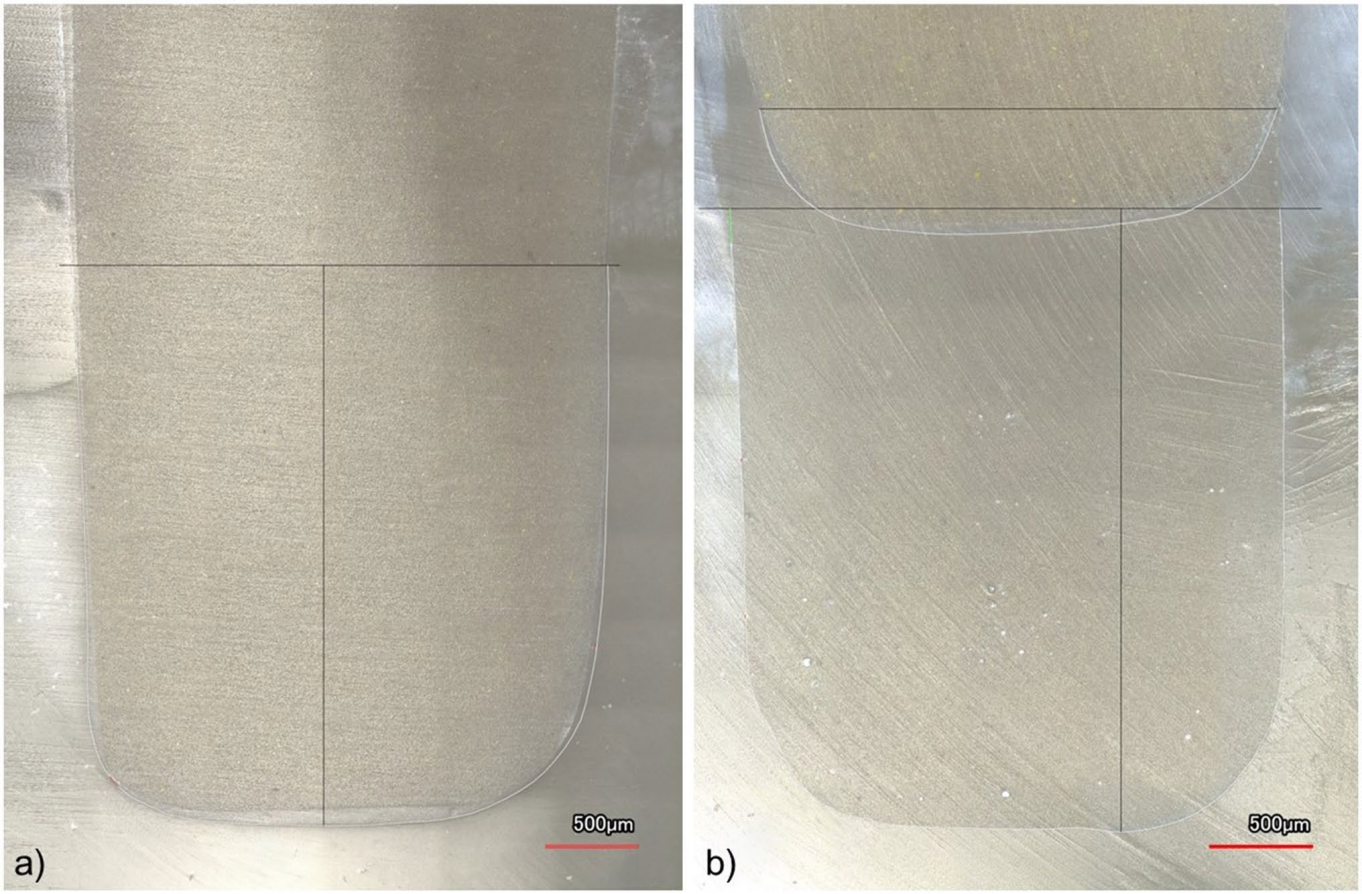
Laser microscope images of the mesial (**a**) and distal (**b**) surfaces of a specimen restored with a PICN inlay after the 2. TML. Standardized guides (black) were drawn in the image analysis software to mark the area in which quantitative analysis was performed. Interfaces 1 and 2 were drawn up to 3 mm from the lowest point of the margin (vertical and lower horizontal line). A fixed width of 2.5 mm (upper horizontal line) marked the ends of interface 3 at the intersection with the inlay margin. Measurement lines were drawn along the entire length of the interfaces, with white, green, and red lines indicating continuous, non-assessable, and non-continuous sections of the restoration margin, respectively

The final quantitative marginal analysis was conducted in the same manner as after the adhesive luting of the inlays. Figure 3, depicting representative images obtained with laser microscopy, describes the assessment method in finer detail.

### Load to fracture

The specimens were loaded until failure in a universal testing machine (Z020, Zwick Roell, Ulm, Germany) to assess the fracture resistance and the fracture mode of the restored teeth. A custom metal holder fixed the specimens in place. To evenly distribute the loading force, a 0.2 mm thick tin foil covered the occlusal surface before a stainless-steel sphere, 4.5 mm in diameter, was positioned on the central fossa of the tooth. An increasing axial compressive load was applied at a crosshead speed of 0.5 mm/min until specimen fracture.

### Fracture analysis

The specimens were examined from five sides (occlusal, buccal, oral, mesial, and distal) under a stereomicroscope (Wild Heerbrugg AG, Heerbrugg, Switzerland) at a magnification of 16 × [8]. A cold light source (DIA Stick, I.C. Lercher, Stockach, Germany) was used to reveal cracks that had not resulted in a tooth fragment breaking off. The fracture lines and crack lines of each specimen were recorded in schematic drawings, all published in an open repository [24]. Each fracture line was assigned to one of the following three categories: (1) fractures affecting exclusively the restoration, (2) fractures affecting both the restoration and the tooth above the simulated bone level, and (3) fractures extending below the simulated bone level [8]. The latter fracture mode was deemed non-restorable, whereas fractures belonging to categories 1 and 2 were considered as restorable [8].

### Statistical analysis

The total length of continuous margin at each interface was presented as a percentage of the respective assessable margin for each interface. To enable data fitting by a conventional distribution function, deviation values (100 - relative continuous value) were calculated. Given the numerous zero (deviation) values, regression models that allowed for overdispersion of the Poisson distribution were performed. For comparisons of repeated measurements, generalized linear mixed models via penalized quasi-likelihood were performed to control within specimen variability. The back transformed regression estimates were geometric mean ratios with the corresponding 95% confidence interval (CI) and *p* value. Owing to the explorative nature of the study, numerous comparisons were performed between various subgroups (analyzed marginal area, restoration material, and proximal surface), combined subgroups, and repeated measurements. Whenever meaningful, pairwise comparisons were calculated by the Tukey contrast adjusting the significance level. Otherwise, comparisons with a reference group were performed without adjusting the significance level. A *p* value ≤ 0.05 was considered as significant. For descriptive statistics, mean and standard deviation (SD) were indicated (rather than median and interquartile range), since the mean ratios of given subgroups coincided well to the corresponding geometric mean ratios derived from the regression models. For descriptive statistics of fracture load, mean and SD were indicated with the corresponding *p* values derived from *t* and *F* tests. Comparison to a reference group were calculated using linear regressions providing difference of mean values, 95% CI, and the corresponding *p* value. Applying the Tukey contrast, multiple comparisons were calculated. Frequency and proportion of fracture mode were indicated with the corresponding *p* values derived from chi-squared or Fisher’s exact tests. A *p* value ≤ 0.05 was considered as significant. An open repository holds the dataset generated and analyzed in the study [24]. A statistician performed all analyses using R software (version 3.5.1, R Core Team, R Foundation for Statistical Computing, Vienna, Austria).

## Results

### Marginal quality

No debonding failure occurred throughout the experiment. All specimens were available for the final quantitative marginal assessment and fracture analysis. The descriptive results of the marginal quality assessments and the results of the multiple regressions are shown in Table 1 and Table 2, respectively.

**Table 1:** Results of the marginal quality assessment. Data are presented as mean of the non-continuous margin with the corresponding SD given in parentheses. The number of specimens with 100% of continuous margin in each assessment group is given in square brackets

	Group, inlay material, number of rounds of TML			
Interface	G1-PICN-2TML	G2-LDS-2TML	G3-PICN-1TML	G4-LDS-1TML
DME, baseline, tooth-RBC	1.02% (2.92) [8/12]	0.10% (0.33) [10/12]	2.94% (7.64) [8/12]	1.99% (2.61) [6/12]
DME, 1.TML, tooth-RBC	1.76% (3.87) [5/12]	0.16% (0.42) [9/12]	N/A	N/A
DME, 2.TML, tooth-RBC	1.80% (3.90) [5/12]	1.31% (3.42) [8/12]	2.96% (7.65) [8/12]	2.15% (2.77) [6/12]
DME, baseline, RBC-inlay	8.25% (9.93) [3/12]	8.53% (11.95) [7/12]	3.32% (6.59) [9/12]	3.68% (12.74) [11/12]
DME, 2.TML, RBC-inlay	9.11% (10.62) [3/12]	8.78% (12.21) [7/12]	3.48% (6.91) [8/12]	3.77% (13.05) [11/12]
No DME, baseline, tooth-RBC	0.43% (0.61) [7/12]	0.97% (2.93) [7/12]	1.38% (3.82) [7/12]	0.09% (0.17) [8/12]
No DME, 1.TML, tooth-RBC	0.57% (0.85) [7/12]	1.00% (2.97) [7/12]	N/A	N/A
No DME, baseline, tooth-inlay	10.26% (14.91) [3/12]	1.37% (3.66) [8/12]	3.09% (4.01) [3/12]	5.58% (10.12) [7/12]
No DME, 2.TML, tooth-inlay	10.94% (15.72) [3/12]	1.45% (3.80) [8/12]	3.35% (4.43) [3/12]	5.78% (10.49) [7/12]

*DME*, deep margin elevation; *LDS*, lithium disilicate; *N/A*, not applicable; *PICN*, polymer-infiltrated ceramic network material; *RBC*, resin-based composite; *TML*, thermomechanical loading

**Table 2:** Results of the multiple regressions

Parameter		Comparison	GMR	95% CI [LL, UL]	*p* value
	Material/interface				
Aging of the DME	PICN/tooth-RBC	non-aged DME vs. aged DME, before 2.TML	1.67	[0.36, 9.52]	0.519
		non-aged DME vs. aged DME, after 2.TML	1.64	[0.36, 9.17]	0.531
	PICN/RBC-inlay	non-aged DME vs. aged DME, before 2.TML	0.40	[0.05, 2.01]	0.301
		non-aged DME vs. aged DME, after 2.TML	0.38	[0.05, 1.80]	0.260
	LDS/tooth-RBC	non-aged DME vs. aged DME, before 2.TML	12.66	[0.67, > 1000]	0.247
		non-aged DME vs. aged DME, after 2.TML	1.64	[0.27, 13.10]	0.593
	LDS/RBC-inlay	non-aged DME vs. aged DME, before 2.TML	0.43	[0.06, 2.04]	0.319
		non-aged DME vs. aged DME, after 2.TML	0.43	[0.06, 1.99]	0.311
	Interface				
Material	Tooth-inlay	PICN vs. LDS, baseline	1.92	[0.64, 6.65]	0.264
		PICN vs. LDS, 2.TML	1.98	[0.68, 6.64]	0.233
	RBC-inlay	PICN vs. LDS, baseline	0.95	[0.33, 2.66]	0.917
		PICN vs. LDS, 2.TML	1.00	[0.37, 2.75]	0.994
	Loading cycle				
Interfaces	Baseline	tooth-inlay vs. tooth-RBC	3.36	[1.29, 10.62]	0.023
		tooth-inlay vs. RBC-ceramic	0.85	[0.40, 1.79]	0.676
	2. TML	tooth-inlay vs. tooth-RBC	2.62	[1.11, 6.98]	0.039
		tooth-inlay vs. RBC-inlay	0.86	[0.41, 1.78]	0.677
	Interface/DME				
2.TML	Tooth-RBC/aged DME	2.TML vs. 1.TML, LDS	8.33	[1.45, 47.62]	0.024
		2.TML vs. baseline, LDS	13.16	[1.52, 111.11]	0.026
		2.TML vs. 1.TML, PICN	1.03	[0.51, 2.06]	0.944
		2 TML vs. baseline, PICN	1.76	[0.78, 3.95]	0.188
	Tooth-RBC/non-aged DME	2.TML vs. baseline, LDS/PICN	1.04	n.e	0.956
	RBC-inlay/aged and non-aged DME	2.TML vs. baseline, LDS/PICN	1.06	[0.61, 1.84]	0.843
	Tooth-inlay	2.TML vs. baseline, LDS/PICN	1.01	[0.56, 1.85]	0.969

*CI*, confidence interval; *DME*, deep margin elevation; *GMR*, geometric mean ratio; *LDS*, lithium disilicate; *LL*, lower limit; *n.e.*, statistically not estimable; *PICN*, polymer-infiltrated ceramic network material; *RBC*, resin-based composite; *TML*, thermomechanical loading; *UL*, upper limit

Whether the RBC left as DME was artificially aged beforehand had no influence on the marginal quality at the interface between the RBC and the tooth and the RBC and the inlay (*p* ≥ 0.247).

At baseline and after the second TML, the restoration material (LDS vs. PICN) had no significant impact on the marginal quality of the inlays (*p* ≥ 0.233).

A higher percentage of non-continuous margin was observed at the interface between the inlay and the tooth compared with the interface between the tooth and the RBC at baseline (*p* = 0.023). Likewise, after the second TML, the marginal quality at interface between the inlay and the tooth showed a higher percentage of non-continuous margin than the interface between the RBC and the tooth (*p* = 0.039). At baseline and after the second TML, the marginal quality at the interface between the inlay and the tooth and at the interface between the inlay and the RBC showed no significant difference (*p* ≥ 0.676).

In specimens with LDS inlays placed on RBC that had previously undergone artificial aging, the second TML led to an increase in the percentage of non-continuous margin at the interface between the RBC and the tooth (*p* ≤ 0.026). In specimens with PICN inlays placed on RBC that had previously undergone artificial aging, no significant change in the marginal quality was observed at the interface between the RBC and the tooth at any time point (*p* ≥ 0.188). The second TML did not significantly affect the marginal quality at the interface between the tooth and the RBC that had not undergone artificial aging beforehand (*p* = 0.956). No significant changes were detected after the second TML at the interface between the inlay and the RCB left as DME and the interface between the inlay and the tooth (*p* ≥ 0.843).

### Load to fracture

The age of the DME had no significant effect on the fracture resistance of the specimens (*p* = 0.052 for LDS, *p* = 0.473 for PICN). Higher fracture loads were recorded in teeth with PICN inlays compared with LDS inlays (*p* < 0.001). Detailed results are reported in Table 3. All specimens exhibited multiple fractures and cracks. Across groups, fracture mode 2 (i.e., fractures affecting both the restoration and the tooth above the simulated bone level) occurred most frequently, followed by fracture mode 3, catastrophic fractures extending below the bone level. Fractures affecting exclusively the restoration were rare. Neither the restoration material nor the age of the DME had a significant impact on the fracture behavior (*p* ≥ 0.149).

**Table 3:** Results of the load to fracture test in the four experimental groups: mean load capability values in *N*, standard deviation (SD), and number of specimens displaying a certain fracture mode

Group	Mean load capability (SD)	Fracture mode 1 (within the restauration)	Fracture mode 2 (restoration and tooth, above bone level)	Fracture mode 3 (catastrophic; below bone level)
G1-PICN-2TML	1953.2 (483.4)	1	28	16
G2-LDS-2TML	1596.9 (385.8)	1	21	19
G3-PICN-1TML	1840.0 (360.7)	1	24	20
G4-LDS-1TML	1290.9 (237.6)	3	23	15

*LDS*, lithium disilicate; *PICN*, polymer-infiltrated ceramic network material; *SD*, standard deviation; *TML*, thermomechanical loading

## Discussion

This laboratory study assessed whether bonding a CAD-CAM inlay on a previously aged direct RBC restoration, used as proximal DME, would have any effect on marginal quality and fracture behavior. The results showed that artificial aging of the RBC restoration to be left for DME had no significant influence on the marginal quality at the interface between the RBC and the inlay. Across time points, the interface between the RBC and the tooth showed a higher percentage of continuous margin compared with the interface between the inlay and the tooth. The fracture behavior and fracture resistance, higher for teeth with PICN than LDS inlays, were not significantly impacted by the age of the DME.

The present study demonstrated no detrimental impact of DME, aged or not, on the marginal quality of the subsequently placed inlay restorations. This corroborates the findings of previous laboratory studies [7, 9, 13]. The interface between DME and the tooth showed fewer discontinuities compared with the interface between the inlay and the tooth, which was probably because impression taking and adhesive luting of indirect restorations are fraught with major challenges in teeth with deep proximal boxes [1]. This study therefore suggests that, in terms of marginal quality, it may be beneficial to restore subgingival proximal boxes with DME made with RBC rather than indirect restorations.

Considering that artificial aging of the RBC restoration in the first run of TML did not impair the quality of the DME, this study, moreover, indicates that it may not be imperative to place a direct RBC restoration for DME in the same visit or shortly before the provision of an indirect restoration. Rather, in select cases where an intact RBC restoration has been in place for some time, it seems acceptable to selectively leave RBC in deep proximal boxes for DME before an indirect restoration is delivered. Thus, the complexity and cost of the treatment can be reduced.

Careful case selection, however, is key when one faces the decision whether to replace a direct RBC restoration or to selectively leave it in place as DME prior to an indirect restoration. The isolation of the working field, a matrix that tightly seals the margin of the subgingival defect, and precision during all working steps involved in the bonding and buildup procedure are essential for DME [1]. When conventional RBC is used, metal matrixes—like the ones applied in the present study—are superior to transparent matrixes, and adequate light polymerization, including light curing from three sides after matrix removal, needs to be ensured [25]. Such details of the restorative procedure of a direct RBC must be taken into consideration along with clinical and radiographic indicators of restoration integrity when one assesses the suitability of an existing RBC restoration for DME. Comprehensive guidance on restorative patient care that is underpinned by scientific evidence and the key tenets of minimally invasive dentistry can be found elsewhere [26].

The age of the DME had no significant impact on the fracture resistance of the restored teeth in the present study. This is in line with data of a systematic review and meta-analysis, which found no significant effect of DME on the fracture resistance of partial indirect restorations [27]. However, in specimens with LDS inlays placed on DME restorations that had been artificially aged beforehand, the subsequent TML reduced the marginal quality at the interface between the RBC and the tooth. By contrast, no such change occurred in specimens with PICN inlays. The implications of this finding are twofold. First, it indicates that long-running TML may cause marginal quality deterioration of DME restorations, which is in accordance with previous research [7, 12, 14]. Second, it suggests that DME restorations under LDS inlays are more susceptible to the cumulative impact of repeated TML than PICN inlays. This is likely due to PICN, with its lower elastic modulus (37.8 GPa), attenuating loading stress transmitted to the DME more effectively than LDS with its elastic modulus of 102.7 GPa [8, 28, 29]. However, one should consider that the proximal dimensions of the inlay matched those of the subjacent DME in the present study, which facilitated stress transfer from the occlusal surface to the DME. In comparison, teeth restored with onlays exhibit a more favorable stress distribution in cervical dentin [28]. Further studies are therefore needed to determine whether stress distribution patterns are indeed more adverse for DME restorations under inlays compared with onlays [30].

LDS and PICN are commonly used to fabricate inlays and onlays, which is why these CAD-CAM materials were selected for the present investigation [31]. Given that materials used for indirect restorations differ in their mechanical behavior, including elasticity, fracture resistance, and force transmission, more research is needed to validate the findings of this study with other materials such as fine-structure feldspar ceramics and CAD-CAM resin-based composites with dispersed fillers [31].

The parameters that influence the adaptation of CAD-CAM restorations are legion and are discussed in great detail elsewhere [32]. It is noteworthy that the marginal quality of LDS and PICN inlays showed some differences at baseline. However, these differences were not statistically significant and a comparison of the internal and marginal fit of the inlays was not within the scope of the investigation.

The appropriate surface pretreatment of RBC left for DME is crucial to enhance the bonding performance of the restoration placed on top of it. In the present study, RBC restorations were reduced with diamond burs before the surface was grit blasted and a universal adhesive was applied. This is a reliable protocol for the repair of direct RBC restorations in defects involving tooth substance and RBC [33]. Arguably, the same principles apply when bonding an indirect restoration on an existing direct RBC restoration. However, data of a recent systematic review and meta-analysis indicate that applying a nonhydrolyzed silane coupling agent as an additional step prior to the adhesive improves the repair bond strength of direct RBC restorations [34]. Though the interface between the RBC and the inlay showed no signs of marginal quality deterioration over time in the present study, the implementation of a nonhydrolyzed silane coupling agent in the pretreatment protocol may prove advantageous for the stability of this adhesive interface in the longer term.

The selection of the adhesive and restoration material for the DME is an important factor for its marginal adaptation [1]. Yet, there is currently no consensus regarding the material of choice for DME [1]. The present study used a three-step etch-and-rinse adhesive and a conventional nanohybrid RBC, applied in layers, to restore the deep proximal boxes because these materials have a proven performance record [35]. The results of the present study are therefore not applicable to materials such as glass ionomers, resin-modified glass ionomers, and flowable RBC, which some dental practitioners and researchers use for DME.

Data of a recent laboratory study suggest that bulk fill RBC with more efficient photo initiators achieves a better marginal quality in proximal boxes below the CEJ compared with conventional RBC [25]. Accordingly, bulk fill RBCs promise to be an equivalent or superior alternative to conventional RBCs even though the marginal quality of DME restorations made with conventional RCB was rated favorably at every time point in the present study. Furthermore, some researchers recommend adhesives that can be applied in self-etch mode or with selective enamel etching rather than etch-and-rinse adhesives because the latter, used in the present study for DME, entail the risk of over-etching the dentin substrate in subgingival areas [1].

This in vitro study only partially took account of the factors that may affect the quality of restorations delivered in clinical practice. The restorative procedures were conducted in ideal conditions, without any patient-related factors affecting them. For instance, the absence of neighboring teeth and soft tissues facilitated digital impression taking in deep marginal boxes, excess removal, and finishing and polishing of the restoration margins. Moreover, the fact that the study did not include a group of teeth restored exclusively with inlays extending to the cervical level in both proximal boxes and that the force used to insert inlays was not strictly standardized must be considered as important limitations. In addition, while based on an established protocol that combines cyclic loading within physiological limits and simultaneous thermocycling [8], artificial aging through thermomechanical loading is a highly simplified simulation of the factors that have a bearing on the longevity of dental restorations in the complex oral environment. This is especially true for chemical and biological factors in the oral cavity, which greatly affect clinical longevity of dental restorations. Consequently, the inherent methodological limitations of this laboratory study must be considered when drawing careful conclusions for clinical practice.

Further investigations should evaluate whether RBC restorations with extended clinical service may be suitable for DME without any unfavorable effect on the long-term performance of the DME or the restoration placed thereon. To gain insight into restoration quality beyond visible restoration margins, methods using X-ray microcomputed tomography are particularly promising [17, 36]. Moreover, clinical studies are required to validate the findings of this laboratory study and to assess patient-centered outcomes of this restorative treatment approach.

## Conclusions

Within the limitations of this in vitro study, it can be concluded that artificial aging of RBC restorations subsequently used as DME had no negative effect on the marginal quality, fracture resistance, and fracture behavior of LDS and PICN inlays. Thus, this investigation suggests that—in select cases—intact, direct RBC restorations that were placed some time before the provision of an indirect restoration may be partially left in place to serve as DME in teeth that require an indirect restoration.

## Data Availability

An open repository holds the dataset generated and analyzed in the study [24].
